# Sleep duration in adolescence buffers the impact of childhood trauma on anxiety and depressive symptoms

**DOI:** 10.1186/s12889-025-21621-x

**Published:** 2025-02-04

**Authors:** Toni Coote, Emma Barrett, Lucinda Grummitt

**Affiliations:** 1 The Matilda Centre for Research in Mental Health and Substance Use, The University of Sydney, Level 6, Jane Foss Russell Building, Sydney, NSW 2006 Australia; 2 Sydney School of Public Health, The University of Sydney, Sydney, NSW Australia

**Keywords:** Mental health, Trauma, Sleep, Anxiety, Depression, Adolescence

## Abstract

**Background:**

Childhood trauma is a pervasive issue contributing to adverse mental health outcomes. Obtaining optimal sleep supports healthy development and protects against mood-related disorders. Whether sleep serves as a potential buffer between trauma and adverse mental health outcomes holds promise for informing targeted interventions and prevention for adolescents.

**Methods:**

Data were drawn from the baseline assessment of a randomised controlled trial of a mental health prevention program. A total sample of 752 adolescents completed an online, self-report survey in 2023. Participants were students (M_age_=13.8 years), attending independent schools in Australia and comprised of 37% girls and 60% boys. Australian sleep guidelines were used to dichotomise nightly sleep duration into whether adolescents met, or did not meet, the sleep guidelines for their age. Mixed-effects linear regression was used to examine whether sleep moderated the association between trauma and symptoms of anxiety, depression, and mental wellbeing.

**Results:**

The majority of participants (82%) reported exposure to at least one traumatic event. The mean number of traumatic events was 1.8. Trauma was independently associated with higher depressive and anxiety symptoms and lower mental wellbeing scores. Those reporting exposure to one or more traumatic events were more likely to report difficulties falling asleep and less likely to report meeting nightly sleep duration guidelines. We found a significant interaction between meeting nightly sleep duration guidelines and any trauma exposure on depressive and anxiety scores, such that depression and anxiety symptoms were lower in trauma-exposed adolescents who met sleep duration guidelines compared to those who did not meet sleep guidelines.

**Conclusions:**

Obtaining optimal amounts of sleep each night may help mitigate anxiety and depressive symptoms for non-clinical adolescents exposed to trauma, however, longitudinal research is needed to confirm the directionality of the relationships between trauma, sleep, and mental health symptoms. Future research should examine the effectiveness of public health interventions targeting sleep behaviours in adolescents to promote mental wellbeing.

**Supplementary Information:**

The online version contains supplementary material available at 10.1186/s12889-025-21621-x.

## Introduction

Over a third of young Australians, aged 16–24, have experienced a mental health disorder in the last 12 months [1]. Globally, mental illness is the leading cause of years of healthy life lost due to disability [2]. Adolescence not only marks a crucial period of biological and social development but also coincides with the peak onset of such mental health disorders [3]. Therefore, adolescence presents an opportune time to effectively address risk factors associated with the development of adverse mental health outcomes and prevent poor mental health trajectories.

### Childhood trauma

Childhood trauma has been evidenced as a major contributing factor in the development of mental ill-health, including depression and anxiety [4, 5]. This is of particular concern with previous studies finding 41% of Australian adults reporting exposure to a traumatic event, such as physical or sexual violence, serious accidents, or natural disasters, before the age of 17 [6]. A recent meta-analytic study on childhood maltreatment, a common cause of trauma in young people including abuse and neglect, estimated up to 21% of depressive disorders and 24% of anxiety disorders in Australia can be attributed to maltreatment during childhood [7]. Experiencing trauma in childhood or adolescence can be particularly harmful as ongoing development is disrupted, causing enduring effects across multiple domains, including social, academic and emotional functioning [8–11]. Exposure to trauma may result in adaptive modifications such as increased threat detection and hypervigilance [12]. However, the persistence of these adaptations beyond the context in which the trauma occurs may be considered maladaptive and in turn heighten susceptibility for anxiety and depressive disorders [13, 14]. Given the high prevalence and detriment to health outcomes attributed to childhood trauma there is a strong public health imperative to address these growing concerns [15]. Therefore, understanding potential intervention targets to prevent mental ill-health in adolescents exposed to trauma is a critical secondary prevention approach.

### Sleep in adolescence

A wealth of literature has demonstrated the critical role of sleep in the development of cognitive functions and mental health [16]. Both insufficient and excessive amounts of sleep have been associated with higher risk for poor mental health outcomes, including in adolescents [17–19]. Sleep and circadian rhythm disruption have been posited as pivotal factors preceding the onset of both anxiety and depression [20–22]. Notably, persistent insomnia has been identified as a causal mechanism in the occurrence of mental health problems in young adults [23]. Additionally, in non-clinical populations, experimental studies have shown sleep duration causally effects mood states in adolescents [24, 25]. Furthermore, sleep difficulties have been associated with more complex mental health presentations in adolescents [26] and linked to suicidality and self-injury [19, 27, 28]. Importantly, those with sleep disturbances are significantly more likely to have mood and psychotic disorders onset during adolescence or early adulthood [29]. While bidirectional relationships between sleep disturbances and mental health symptoms have been evidenced [30, 31], it is unclear whether this reflects a common aetiology or if sleep disturbances precede and subsequently aggravate mutually reenforcing factors. For example, hyperarousal models of insomnia posit cognitive processes, such as worry and rumination, as both consequence of and an exacerbating factor of sleep reactivity [32]. Furthermore, many of the factors implicated in the biopsychosocial model of sleep and mental health overlap, including high-risk genetic variances [33, 34]. Such entanglement complicates distinguishing a clear causal pathway. However, across biological, psychological and social domains evidence increasingly indicates that sleep disturbances may underly the development of mental health disorders [34]. These findings together underscore the importance of adolescence as a crucial period for healthy sleep. However, an Australian study found only 20% of adolescents were obtaining optimal amounts of sleep [35]. In light of this, in 2018, the Australian government supported renewed age-based guidelines incorporating physical activity, sedentary behaviour and sleep across the 24-hour period for young people including adolescents [36, 37], hereinafter, Australian sleep duration guidelines. The Australian sleep duration guidelines were developed utilising evidence informing Canadian guidelines in conjunction with an updated systematic review [37]. Such recommendations are agreed upon across Australia, Canada and United States [38, 39].

### Sleep and trauma

Importantly, trauma has been associated with sleep disturbances in adolescents [40, 41], persisting through to adulthood [42, 43]. Several mechanisms have been proposed to explain the way in which trauma affects sleep including physiological hyperarousal and cognitive hyperactivation [44]. Exposure to a traumatic event has been posited to induce and maintain states of physiological hyperarousal, which may interfere directly with sleep while increasing susceptibility to other mental health conditions, such as anxiety and depression [45, 46]. Disrupted sleep-wake cycles, or circadian dysregulation, have been shown to further exacerbate physiological sensitivity to stress [47, 48]. When prolonged, this can lead to chronic psychological stress and maladaptation [47, 48]. Thus, sleep has been posited as a core component in the pathophysiology of trauma-related disorders including post-traumatic stress disorder (PTSD) [47] and a prognostic factor in the development of depression and anxiety [49]. While physiological hyperarousal has been linked to depression and anxiety disorders, the role cognitive and emotional processes play, and the exact neurological mechanism remains an area for further research [44, 50–52]. Therefore, whether sleep may serve to buffer against poor mental health in adolescents exposed to trauma remains unclear. Clarifying this may help elucidate whether addressing sleep disturbances may help to disrupt the physiological sequelae following trauma exposure and protect against adverse mental health outcomes for adolescents exposed to trauma.

### The current study

There is little research examining these relationships in early adolescence, despite the importance of this period for both the development of healthy sleep practices and the emergence of mental health symptoms. Many studies have examined bivariate associations in late adolescence or adolescence more broadly [24, 25, 41], which may preclude identifying developmental periods most amenable to behavioural interventions. Existing studies have investigated associations between trauma, sleep and mental health in clinical, young adult or adult populations [53–55]. The generalisability of these results to early adolescent populations remains unclear. Investigating sleep as a moderator between trauma exposure and mental health may help to identify adolescents at heightened risk for poor mental health, thereby informing targeted prevention efforts. Therefore, this study aims to investigate associations between sleep, trauma and mental health and whether sleep moderates the relationship between trauma and mental health outcomes in an Australian adolescent sample. Specifically, we first aimed to observe whether any exposure to trauma was independently associated with mental health outcomes. We hypothesised that adolescents reporting one or more exposures to traumatic events would, on average, score higher in depressive and anxiety symptoms and lower in mental wellbeing. Second, we aimed to examine whether trauma was associated with sleep. We hypothesised that those with exposure to trauma would be more likely to report difficulties falling asleep and less likely to meet Australian sleep duration guidelines. Lastly, we aimed to investigate whether sleep moderates the impact of trauma on mental health. Specifically, we hypothesised that sleep would have a significant interaction with trauma, such that participants exposed to trauma with adequate sleep duration would show better mental health outcomes compared to their trauma-exposed peers with sub-optimal sleep duration.

## Methods

The current study drew on baseline data collected as part of the OurFutures Mental Health study, an ongoing cluster randomised trial evaluating the effectiveness of an online mental health intervention for Year 8 and 9 students (approximately 13 years of age). As described by Grummitt and colleagues (2023) in the pre-registered protocol, from 2022 to 2023 independent schools Australia-wide were contacted through publicly available contact details and invited to participate in the study [56]. Schools that agreed to participate were block randomised to intervention or control groups and information and consent forms were sent to parents. Students who were not opted-out of participating in the study by a parent or guardian provided active, informed consent before completing an online, self-report survey, during class time. No additional exclusion criteria were applied. Aboriginal and Torres Strait Islander status was not collected due to ethical considerations. Ethical approval was granted from independent (non-government) schools, while the government schools’ ethics approval body did not allow for data on trauma exposure to be collected. Thus, the current sample includes independent schools only and excludes one government school that participated in the trial but did not collect data relevant to the current study. The current study analysed a total sample of 752 Year 8 and 9 students attending one of nine participating schools across NSW, VIC, SA and QLD who completed the baseline survey. The OurFutures: Mental Health study was registered with the Australian and New Zealand Clinical Trials Registry (ACTRN12622001582741). Ethical approval for this study was obtained from the University of Sydney Human Research Ethics Committee (HREC Approval Number 2022/700).

### Measures

All measures were collected at the baseline assessment, to remove any possible effect of the intervention on the relationships between trauma, sleep, and mental health.

### Trauma

Trauma exposure was measured using the Child Trauma Screen (CTS) [57], a ten-item screening measure for children. Of these ten items, four specifically aim to assess exposure to traumatic events and were used in this study. The four items from the CTS “Events” section, comprising of four yes/no questions, were used to indicate the participants’ exposures to traumatic events, such as ‘Have you ever seen people pushing, hitting, throwing things at each other, or stabbing, shooting or trying to hurt each other?’; ‘Has someone ever touched you on the parts of your body that a bathing suit covers, in a way that made you uncomfortable?’. A dichotomous categorical variable was computed to categorise participants responding ‘No’ to all four events as not having exposure to trauma and ‘Yes’ to one or more of the events as having exposure to trauma. The CTS has been used to screen adolescent inpatients and children and adolescents in welfare custody in the US [58, 59] and further validated in community and primary care clinics [60, 61].

### Sleep

To assess average nightly sleep duration over the past three months, participants were asked to select from nine alternatives (less than 5 h, 5, 6, 7, 8, 9, 10, 11 or more than 11 h); (see Supplementary Material). Responses were categorised as a dichotomous variable reflecting whether reported sleep duration met the Australian sleep duration guidelines according to age. As such, participants aged up to 13 years old reporting 9–11 h of sleep and participants aged 14 years or older reporting 8–10 h of sleep were coded as meeting guidelines; all other responses falling outside the recommended hours according to their age was coded as not meeting guidelines. Self-reported sleep duration has been shown to be an acceptable approximation for adolescents’ sleep duration [62].

Difficulties falling asleep was assessed from a second question asking whether the participant has difficulty falling asleep (yes/no); (see Supplementary Material).

### Mental health

Depressive symptoms over the past two weeks were measured through the Patient Health Questionnaire adapted for Adolescents (PHQ-A) [63]. The PHQ-A has shown strong diagnostic validity and accuracy for adolescents [63]. One item regarding suicidal ideation was removed due to ethical concerns. The eight-item PHQ has demonstrated equivalent validity and accuracy compared to the nine-item version [64]. The eight-item PHQ-A has been used as a measure of depressive symptoms in intervention studies targeting adolescent mental health in Australia and the United States [65, 66]. The eight-item PHQ-A asked participants how often they have been bothered by a range of depressive symptoms (e.g. ‘Feeling down, depressed, irritable, or hopeless?’; Little interest or pleasure in doing things?’). Possible responses for each item ranged from 0, ‘Not at all’, to 3, ‘Nearly every day’. Responses were summed to provide a continuous score with higher scores indicating more severe depressive symptoms. Total scores range from 0 to 24. A total score of *≥* 10 indicates probable cases of current depression [64].

Anxiety symptoms over the past two weeks were measured through the Generalised Anxiety Disorder Screen Tool (GAD-7) [67]. The GAD-7 has been validated as a reliable tool for assessing anxiety symptoms in adolescent populations [68]. The GAD-7 has been used to study associations between childhood trauma and anxiety symptoms in young Australians [69] and in large school-based intervention studies for students in Years 8–10 in Australia [66]. The GAD-7 comprises of seven-items asking participants how often they have been bothered by a range of anxiety symptoms (e.g. ‘Feeling nervous, anxious, or on edge’; ‘Not being able to stop or control worrying’). Possible responses for each item ranged from 0, ‘Not at all’, to 3, ‘Nearly every day’. Responses were summed to provide a continuous score with higher scores indicating more frequent anxiety symptoms. Total scores range from 0 to 21. A total score of > 10 indicates probable cases of current anxiety disorder [67].

Mental wellbeing over the past two weeks were measured through the Short Warwick-Edinburgh Mental Well-being Scale (SWEMWBS) [70]. The SWEMWBS has shown good validity and reliability in adolescents [71]. The SWEMWBS comprises of seven items (e.g. ‘I’ve been feeling optimistic about the future’; ‘I’ve been feeling relaxed’) with five possible responses ranging from 1, ‘none of the time’, to 5, ‘all of the time’. Responses were summed to provide a continuous score with higher scores indicating greater positive mental wellbeing. Total scores on the SWEMWBS range from 7 to 35. In adolescent samples, the SWEMWBS has a mean of 24.6–25.4 and a standard deviation of 4.2–5 [72, 73].

### Statistical analysis

Descriptive statistical analyses were first conducted to obtain participant characteristics across variables. Means and standard deviations were used for continuous variables, depressive symptoms, anxiety symptoms and mental wellbeing. Counts and percentages were used to calculate the prevalence of sleep and trauma measures. Mixed effects linear regression analyses with a random intercept were performed to account for the nested structure of the data (students nested within schools). Analyses were first run without interaction terms for each mental health outcome independently for each trauma and sleep measure serving as the predictor variable while adjusting for age, gender and school clustering. Mixed effects logistic regression models with a random intercept were used to explore associations between trauma exposure and sleep while adjusting for age, gender and school clustering. Finally, to test whether the effect of trauma on mental health outcomes differed depending on sleep duration and difficulties falling asleep, trauma exposure and each measure of sleep was included as an interaction term in separate linear mixed effects models, using a random intercept and adjusting for age, gender and school clustering. Alpha was set at *p* < 0.05. All statistical analysis was performed using R Studio Version 4.3.1. Missing data were low across all included variables (< 10%) and assumed to be missing completely at random, as indicated by a non-significant Little’s MCAR test (*p* = 0.200). Therefore, linear mixed-effects models accounted for missing data through Restricted Maximum Likelihood (REML) estimation through the lme4 package [74]. REML provides estimates using all available data and adjusting for the fixed effects. Thus, estimates presented are derived from the likelihood function of the observed data.

### Sensitivity analysis

Sensitivity analyses were conducted to examine whether the inclusion of a sleep-related item within the measure of depression impacted statistical models involving either sleep measure. To assess potential bias, one item, measuring sleep disturbances, was removed from the PHQ-A measure and results were recalculated for models where either sleep measure was inputted. Second, we assessed the impact of including participants who endorsed witnessing violence as the only trauma exposure by removing one item from the CTS and recalculating results for the models of interaction.

## Results

### Sample characteristics

A total of 752 participants who completed the baseline questionnaire of the OurFutures Mental Health study were included in the present study. The study sample comprised of students attending independent schools across Australia aged 12–16 years old (mean age = 13.8 years). Girls comprised 36.8% of the sample and boys made up 60.1% of the sample. A total of 602 (82.2%) of participants reported exposure to at least one traumatic event. Most students (68%) reported their average nightly sleep duration to be between 7 and 9 h of sleep over the past three months with approximately half (49.9%) of sampled adolescents meeting the Australian sleep duration guidelines according to age. Two in five adolescents (43.3%, *n* = 305) reported difficulties falling asleep. Of the adolescents sampled, approximately one quarter (25.5%) met the GAD-7 threshold for probable anxiety and almost one third (31.5%) met the PHQ-A threshold for probable depression. Participants identifying as boys were more likely to meet nightly sleep duration guidelines compared to females, 57.8% and 39.8%, respectively. Of those identifying as girls, 60.5% reported difficulties falling asleep while only 30.6% of boys reported difficulties falling asleep. The proportion of participants reporting any trauma exposure was similar across gender identity. Complete descriptive statistics for the sample are shown in Tables 1 and 2. Frequencies of reported trauma exposures by type are presented in Supplementary Material Table 1.

**Table 1:** Demographic characteristics of participants in study, *n* = 752

Variable	Value
Age (years), *mean (SD)*	13.8 (0.8)
Gender, *n (%)*	
Boy	452 (60.1)
Girl	277 (36.8)
Non-binary	7 (0.9)
Other term/Prefer not to say	16 (2.1)
Country of birth, *n (%)*	
Australia	687 (91.4)
Other English-speaking country	28 (3.7)
Non-English-speaking country	37 (4.9)

**Table 2:** Clinical characteristics of participants in study, *n* = 752

	Value				
Variable	Boys		Girls		Total
Any trauma exposure, *n (%)*^*a*^					
No	81 (18.6)		47 (17.1)		130 (17.8)
Yes	354 (81.4)		228 (82.9)		602 (82.2)
Number of types of trauma exposure, *n (%)*^*a*^					
0	81 (18.6)		47 (17.1)		130 (17.8)
1	105 (24.1)		67 (24.4)		177 (24.2)
2	123 (28.3)		66 (24.0)		192 (26.2)
3	102 (23.5)		63 (22.9)		170 (23.3)
4	24 (5.5)		32 (11.6)		63 (8.6)
Number of traumatic events, *mean (SD)*^*a*^	1.7 (1.2)		1.8 (1.3)		1.8 (1.2)
Number of traumatic events, *mean (SD)*^*d*^	2.1 (0.9)		2.3 (1.0)		2.2 (1.0)
Meets nightly sleep duration guidelines, *n (%)*^*b*^					
Yes	224 (57.8)		104 (39.8)		352 (49.9)
No	178 (42.2)		157 (60.2)		353 (50.1)
Difficulty falling asleep, *n (%)*^*b*^					
No	293 (69.4)		103 (39.5)		400 (56.7)
Yes	129 (30.6)		158 (60.5)		305 (43.3)
Mental health, *mean (SD)*^*c*^					
Depressive symptoms (PHQ-8)	5.1 (4.6)		10.6 (6.7)		7.3(6.2)
Anxiety symptoms (GAD-7)	4.0 (4.3)		9.3 (6.6)		6.2 (6.0)
Mental wellbeing score (SWEMWBS)	25.1 (5.7)		21.4 (6.1)		23.5 (6.2)
Probable depression, *n (%)*^*c*^					
No	368 (83.3)		131 (47.5)		507 (68.5)
Yes	74 (16.7)		145 (52.5)		233 (31.5)
Probable anxiety, *n (%)*^*c*^					
No	388 (87.8)		152 (55.1)		551 (74.5)
Yes	54 (12.2)		124 (44.9)		189 (25.5)

^*a*^Missing data for 20 participants

^*b*^Missing data for 47 participants

^*c*^Missing data for 12 participants

^d^Among those exposed to any trauma

### Associations with mental health

Mixed effects linear regression models revealed trauma exposure was significantly associated with depressive symptoms (B = 4.08, *p* = < 0.001), anxiety symptoms (B = 3.81, *p* = < 0.001) and mental wellbeing (B = −2.17, *p* = < 0.001), after adjusting for age, gender, and school-level clustering. On average, those reporting any trauma exposure had higher depressive and anxiety symptoms compared to those without trauma exposure (B = 4.08, *p* = < 0.001 and B = 3.81, *p* = < 0.001, respectively). Whereas those reporting any trauma exposure scored lower in mental wellbeing compared to those without trauma exposure (B = −2.17, *p* = < 0.001). Similarly, after adjusting for age, gender, and school-level clustering, not meeting nightly sleep duration guidelines was significantly positively associated with depressive and anxiety symptoms (B = 3.74, *p* = < 0.001 and B = 2.84, *p* = < 0.001, respectively) and significantly negatively associated with mental wellbeing (B = −3.12, *p* = < 0.001). Likewise, difficulties falling asleep was associated with significantly higher depressive symptoms and anxiety symptoms (B = 5.06, *p* = < 0.001 and B = 3.81, *p* = < 0.001, respectively), and significantly lower mental wellbeing (B = −3.01, *p* = < 0.001), after adjusting for age, gender, and school-level clustering. Full results from these models are presented in Table 3.

**Table 3:** Linear mixed effects regression models testing individual associations between trauma and sleep and mental health and well-being outcomes. All models adjusted for school clustering, gender and age

	Depressive symptoms		Anxiety Symptoms		Mental Wellbeing	
	B (SE)	*p*	B (SE)	*p*	B (SE)	*p*
Any trauma exposure	4.08 (0.51)	< 0.001	3.81 (0.49)	< 0.001	−2.17 (0.55)	< 0.001
Does not meet sleep duration guidelines	3.74 (0.41)	< 0.001	2.84 (0.40)	< 0.001	−3.12 (0.43)	< 0.001
Difficulties falling asleep	5.06 (0.40)	< 0.001	3.81 (0.40)	< 0.001	−3.01 (0.44)	< 0.001

After adjusting for age, gender and school clustering, those exposed to trauma were 6.59 times more likely to meet the threshold for probable depression compared to adolescents who did not report trauma exposure, holding all other variables constant (95% CI: 3.46–12.57, *p* = < 0.001). Additionally, we found adolescents with exposure to trauma were 5.55 times more likely to meet the threshold for probable anxiety compared to adolescents without trauma exposure, holding all other variables constant (95% CI: 2.77–11.12, *p* = < 0.001). Results from these models are presented in Table 4.

**Table 4:** Linear mixed effects regression models testing associations between trauma and probable mood disorders. All models adjusted for school clustering, gender and age

	Any trauma exposure	
	Odds ratio (95% CI)	*p*
Probable depression	6.59 (3.46–12.57)	< 0.001
Probable anxiety	5.55 (2.77–11.12)	< 0.001

### Associations between trauma and sleep

A mixed effects logistic regression model was conducted to investigate the difference between meeting nightly sleep duration guidelines for those exposed to trauma and those without, after adjusting for age, gender and school clustering. From this, a significant difference was observed, revealing adolescents exposed to trauma were 1.62 times more likely to obtain suboptimal amounts of sleep compared to adolescents who did not report trauma exposure, holding all other variables constant (95% CI:1.21–2.02, *p* = < 0.001). Similarly, we found a significant difference in trauma exposure groups when examining reports of difficulties falling asleep. We found adolescents with exposure to trauma were 3.15 times more likely to report difficulties falling asleep compared to adolescents without trauma exposure, holding all other variables constant (95% CI: 2.69–3.62, *p* = 0.021). Full results from these models are presented in Table 5.

**Table 5:** Linear mixed effects regression models testing associations between trauma and sleep. All models adjusted for school clustering, gender and age

	Any trauma exposure	
	Odds ratio (95% CI)	*p*
Does not meet sleep duration guidelines	1.62 (1.21–2.02)	0.021
Difficulties falling asleep	3.15 (2.69–3.62)	< 0.001

### Interactions between trauma and sleep measures

**Fig. 1:**
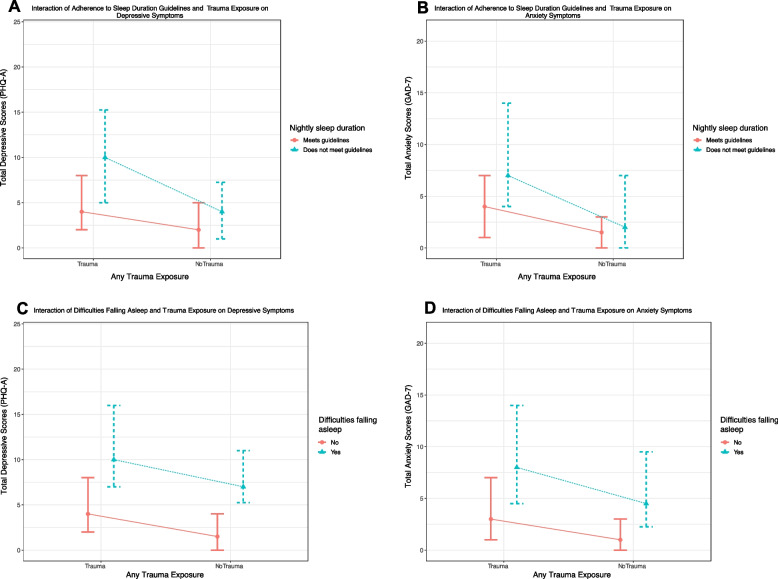
Interaction of childhood trauma and sleep on depressive (left panels) and anxiety (right panels) scores. Top panels show the interaction with childhood trauma and adherence to the nightly sleep duration guidelines; bottom panels show the interaction with childhood trauma and difficulties falling asleep

Analyses revealed that meeting sleep duration guidelines moderated the effect of trauma on some mental health outcomes. After adjusting for gender, age and school clustering, there were significant interaction effects between trauma and meeting sleep duration guidelines on both depressive and anxiety symptoms (*p* = 0.001, *p* = 0.014, respectively). Specifically, the effect of trauma on anxiety and depressive symptoms was greater for those who did not meet the sleep duration guidelines compared to those who did, holding all other variables constant. Participants exposed to trauma who met sleep duration guidelines had significantly lower anxiety and depressive symptoms compared to those who did not meet sleep duration guidelines. No significant interactions emerged between meeting sleep duration guidelines and mental wellbeing. Likewise, we did not find evidence to support difficulties falling asleep moderated effects of trauma on any mental health outcomes. Full results from these models are presented in Table 6 and interaction effects for depressive and anxiety scores are presented in Fig. 1. Additional analyses examining the interaction between number of types of trauma exposure and sleep measures on mental health outcomes are presented in Supplementary Material Table 2.

**Table 6:** Linear mixed effects models individually testing interaction effects between trauma and sleep on mental health and well-being outcomes. All models adjusted for school clustering, gender and age

	Depressive symptoms		Anxiety Symptoms		Mental Wellbeing	
	B (SE)	*p*	B (SE)	*p*	B (SE)	*p*
Intercept	−5.17 (3.69)	0.162	−1.19 (3.62)	0.742	32.85 (4.07)	**< 0.001**
Any trauma exposure	2.31 (0.66)	**< 0.001**	2.53 (0.64)	**< 0.001**	−1.78 (0.71)	**0.013**
Does not meet sleep duration guidelines	0.76 (0.91)	0.405	0.56 (0.89)	0.529	−2.35 (0.99)	**0.018**
Any trauma exposure*Does not meet sleep duration guidelines	3.24 (1.00)	**0.001**	2.41 (0.98)	**0.014**	−0.72 (1.08)	0.506
Intercept	−1.12 (3.51)	0.750	2.00 (3.49)	0.568	28.30 (4.02)	**< 0.001**
Any trauma exposure	2.92 (0.57)	**< 0.001**	2.87 (0.57)	**< 0.001**	−1.61 (0.64)	**0.013**
Difficulties falling asleep	4.20 (1.03)	**< 0.001**	2.71 (1.02)	**0.008**	−2.17 (1.15)	0.060
Any trauma exposure*Difficulties falling asleep	0.44 (1.10)	0.688	0.74 (1.09)	0.498	−0.64 (1.23)	0.602

Bold variables with *p*-value < 0.05

Results of sensitivity analyses are presented in Supplementary Material Tables 3–5. As shown, the interpretation of results did not change from the main analyses presented herein. Repeated analyses excluding one item from the PHQ-A measure showed not meeting nightly sleep duration guidelines and difficulties falling asleep remained significantly positively associated with depressive symptoms, (B = 3.00, *p* = < 0.001, B = 3.73, *p* = < 0.001, respectively). Notably, there was a modest reduction in the effect size of the association between difficulties falling asleep and depressive symptoms compared with results using the eight-item PHQ-A. A significant interaction remained between any trauma exposure and not meeting sleep duration guidelines on depressive symptoms (*p* = 0.002). Likewise, the interaction between any trauma exposure and difficulties falling asleep on depressive symptoms remained non-significant (*p* = 0.151). Additionally, interaction models excluding witnessing violence from the trauma measure, showed similar findings as presented in the main analyses. A significant interaction remained between any trauma exposure and not meeting sleep duration guidelines on depressive and anxiety symptoms (*p* = < 0.001, *p* = < 0.001, respectively). No other interaction was found to be significant.

## Discussion

In a large sample of Australian adolescents, this study found that trauma was independently associated with higher levels of depressive and anxiety symptoms and lower mental wellbeing. As hypothesised, we found adolescents who reported exposure to one or more events were significantly more likely to report difficulties falling asleep and obtain sub-optimal amounts of sleep according to Australian sleep duration guidelines. Importantly, not meeting sleep duration guidelines was found to moderate the relationship between childhood trauma and anxiety and depressive symptoms. Notably, in our non-clinical sample, we found 82% of surveyed adolescents experienced exposure to at least one traumatic event.

These findings predominately aligned with our hypotheses. We found evidence to suggest that not adhering to nightly sleep duration guidelines may exacerbate the negative effects of trauma exposure on anxiety and depressive symptoms for adolescents, after controlling for age and gender. However, we did not find any significant moderating effects between trauma and sleep duration on mental wellbeing. This is surprising, as sleep duration has been shown to have a greater effect on positive affect compared to negative affect [24, 31]. It is possible that subtypes of positive affect reflect high states of arousal and thereby may contribute to sleep disturbances [75]. Moreover, while self-reported difficulty falling asleep was found to be a significant predictor of anxiety and depressive symptoms, contrary to our hypothesis, we did not find significant interaction effects with trauma on mental health outcomes, suggesting difficulties falling asleep may be associated with mental health of adolescents irrespective of trauma exposure.

A substantially larger proportion of students reported exposure to one or more traumatic events compared to a recent study of Australians aged 16 years or older which found just over 60% experienced one or more types of childhood maltreatment [76]. Consistent with previous Australian studies [77], we found half (50%) of participating adolescents obtained optimal amounts of nightly sleep accordingly to Australian sleep duration guidelines. Likewise, we found a point prevalence of reported difficulties falling asleep to be 43%, within the range of 30–50% produced in other Australian adolescent samples [31, 35, 77]. Our study findings align with previous work highlighting significant bivariate associations between trauma, sleep and mental health [78–80]. We found similar interaction effects to those found in examining the relationship between sleep and psychosocial stressors on mental health outcomes in children and adolescents [81].

### Implications and future directions

This study addresses important gaps in the existing literature by investigating the relationship between adherence to nightly sleep duration guidelines and trauma on the association with adverse mental health outcomes in adolescents. This study supports the growing evidence on the importance of sleep in the prevention of adverse mental health outcomes among adolescents exposed to trauma. Additionally, by way of associations with mental health, this study provides support for the Australian sleep duration guidelines for children and young people.

While causal direction cannot be explicitly inferred from the current study, the current findings contribute to the understanding of nightly sleep duration as an important moderator in the development of depressive and anxiety symptoms in adolescents exposed to trauma. Consideration should therefore be given to interventions targeting sleep duration in adolescents exposed to trauma. Sleep interventions vary widely from psychoeducation, behavioural, psychological and psychopharmacological approaches [82]. Much of sleep intervention research has focussed on Cognitive Behavioural Therapy for Insomnia (CBT-I) which has been evidenced as effective in treating comorbid psychiatric disorders in adults [83]. While the applicability of CBT-I in school-aged individuals calls for further investigation, existing literature supports its potential utility in treating insomnia, depression and anxiety in adolescent populations [84, 85].

On a population scale, a promising line of inquiry has highlighted the influence of modifiable elements of the built and social environment on mental health [86, 87]. For example, light exposure has been increasingly shown as an important factor associated with sleep [88], psychotic disorders in adults [89] and mood disorders in adolescents [90]. Similarly, while biological changes during adolescence contribute to delayed sleep-wake cycles [16], sleep-competing and compromising behaviours such as early school start times can undermine adolescents’ mental health by preventing optimal sleep duration [87, 91]. In addition, homework and technology use have been implicated as factors deterring adolescents from obtaining optimal amounts of sleep [77, 92, 93]. This study aligns with the mounting evidence characterising circadian disruption, most accessible through sleep behaviours, as a core component in the pathophysiology of trauma-related disorders [47]. Additionally, several empirical studies have supported the conceptualisation of sleep disturbance as a transdiagnostic risk factor for several mental health disorders [94, 95]. Therefore, interventions targeting modifiable extrinsic factors disrupting sleep-wake cycles may present an effective yet accessible strategy for adolescents exposed to trauma. Examining the effectiveness of sleep-focussed interventions for adolescents exposed to trauma may elucidate whether sleep underlies a critical pathway from trauma exposure to negative mental health outcomes.

Importantly, the relationships evidenced in the current study also underscore the potential value in integrated approaches. For example, concurrent treatment of sleep- and trauma-related psychotherapy outcomes has been shown to be advantageous in adults with PTSD [96]. Given the relationship between sleep and mental health operates and interacts across multiple domains, treatments that combine biological, psychological and social approaches may hold promise for preventing depression and anxiety disorders [34]. Further exploration is therefore warranted to ascertain whether such strategies are effective in the prevention of mental ill-health in adolescents exposed to trauma.

Given the high prevalence of childhood trauma found and its well evidenced impact on health outcomes, there is an urgent need to enhance prevention efforts on a population scale. While universal parenting programs have shown effectiveness in preventing childhood maltreatment, much of the evidence is limited and of moderate quality [97]. It is vital to acknowledge many of the factors that serve to increase the risk of childhood trauma are imposed by inequitable political and social determinants. For example, low socioeconomic status has long been associated with increased risk of maltreatment [98] and an Australian birth cohort study found 27.3% of childhood maltreatment was attributable to economic factors [99]. Given that parental burnout has found to mediate the effect of socioeconomic status on childhood maltreatment [100], it follows that paid parental leave has been shown to lower the risk of infant maltreatment [101]. Together, findings highlight the need to prioritise policy instruments to foster supportive and stable home environments to mitigate parental hardship and effectively reduce childhood trauma.

### Strengths and limitations

The current study collected data on trauma exposure from adolescents (mean age = 13.8). Trauma was assessed using four items from the CTS, a well-supported screening tool showing high validity and reliability with items scoring exposure to traumatic events derived from The Diagnostic and Statistical Manual of Mental Disorders, Fifth Edition criterion for PTSD [57]. The use of subjective reports of childhood trauma have shown to better predict psychopathology than documented (i.e., through child protective services investigations) but not recalled childhood trauma [102]. Many existing studies estimating trauma exposure during childhood or adolescence have relied on retrospective reports recalled during adulthood or secondary reports on behalf of participants such as reports by parents or teachers [103]. This is an important distinction as methods of data collection on trauma exposure have shown to produce significant differences in prevalence estimates [104], with parent-report producing underestimates of trauma exposure [105]. It is therefore likely that data collected on trauma exposure in adolescents has been widely underestimated as such, this study provides novel insights by utilising data obtained during adolescence.

Much of the existing literature on trauma and sleep has studied adult populations however, the results found in studies of adults may not be generalisable to adolescent populations due greater sleep needs [106] and physiological brain maturation affecting sleep, mental health and cognitive development [16]. Similarly, of the studies investigating the relationships between trauma, sleep and mental health in adolescents, much of the research has focussed on clinical populations. By examining this relationship in a non-clinical sample, potential confounds are minimised and results more widely applicable to the general population of adolescents in Australia. Many studies have aimed to elucidate the causal pathway between these variables however, the vast majority have utilised mediation analysis on cross-sectional data [107–109], such findings are subject to inherent and significant bias in both direct and indirect effects [110–112]. Our results highlight that sleep can moderate the impact of trauma on anxiety and depressive scores in non-clinical adolescent populations. Given adolescence is a critical development period during which many mental disorders have their onset [3], findings in this population are better positioned to identify important risk factors for early intervention thereby preventing adverse trajectories and poor long-term mental health outcomes.

Importantly, this study has several limitations to consider which may be addressed in future studies. Due to the cross-sectional design of the present study, causal mechanisms cannot be inferred from the results as all data were collected at a single point in time. As such, it is possible that adolescents with antecedent anxiety and depressive symptoms recalled and subsequently reported prior exposures less favourably than those without. It is also acknowledged that depression and anxiety can cause sleep disruptions, and the cross-sectional nature of the data cannot rule out this reverse causality. While theoretical and longitudinal studies are strongly suggestive of the direction of effects from childhood trauma to sleep to mental health proposed in the current study [34, 79], future research incorporating prospective designs throughout adolescence are needed to confirm the temporal dynamics and directions of such effects.

This study examined the effects of any trauma exposure. As such, specific characteristics of trauma are not accounted for. While a recent review found different types of adverse childhood events to produce similar effects in adult sleep outcomes [113], childhood trauma subtypes have been shown to differentially effect neuroendocrine functioning in youth, which plays an important role in sleep, along with other brain structuring and function [13, 114]. Additionally, variability in trauma exposure, such as timing relative to development, severity and frequency along with other contextual factors, such as social support, were not measured and may contribute to responses to trauma [115, 116]. Furthermore, a recent review found significant heterogeneity across different measures of trauma exposure in adults [117] and thus results must be considered within the distinct context. Similarly, the development of depressive and anxiety symptoms in adolescents is likely influenced by several factors not accounted for within the current study. It is likely genetic predispositions along with pre-and post-natal vulnerabilities interact with social and environmental influences to confer risk of psychopathology onset during adolescence [118].

Average nightly sleep duration was assessed retrospectively, through a self-report questionnaire which was subject to participants’ memory and potential social desirability bias. The measure used in this study asked for a single response aggregating average nightly sleep duration over the past three months. While subjective reports of sleep duration have been shown as an acceptable approximation of objective sleep duration for adolescents, including those with mental ill-health, in clinical settings [62, 119, 120], Australian students have been found to overestimate sleep duration when compared to actigraphy [121]. Therefore, effects of sleep duration as reported in this study must be considered in light of absence of comparable objective measures. However, this study suggests that among adolescents, even imprecisely assessed adherence to nightly sleep duration guidelines may be a considerable moderator for the impact of trauma exposure on anxiety and depressive symptoms. As such, further research utilising more accurate measures of sleep may evidence an even greater influence of sleep duration on the impact of trauma on mental health. Due to prior work finding a positive relationship between both insufficient and excessive hours of sleep and adverse mental health outcomes [17–19], sleep duration was dichotomised. However, given trauma is correlated with insomnia but not hypersomnia [108, 109], it is possible that the inclusion of a dichotomous sleep duration variable may have resulted in some information loss. Although Australian sleep duration guidelines are informed by the best available evidence [36, 37], the literature specific to adolescents remains limited and methods used to derive specific recommendations, including age group categorisation, lacks details and replicability, largely relying on expert consensus [18, 37, 38]. Additionally, difficulties falling asleep was measured through one self-report item. The findings of the current study did not support the hypothesis of a moderating effect of difficulties falling asleep on trauma on mental health despite a significant main effect when independently examined. This may have been due to the subjective nature of the individual item used. Moreover, the item captured specifically difficulties falling asleep. It is possible that sleep difficulties more broadly, including waking frequently after falling asleep, nightmares, or other sleep disturbances are more important factors in the relationship between trauma and mental health outcomes [80]. Furthermore, there were slight variations in response rates observed across the trauma, sleep and mental health measures, which may be attributable to the survey length, leading participants to disengage or time constraints within the classroom. Future studies may benefit from use of validated measures of sleep duration and difficulties for adolescents.

Lastly, while schools invited to participate were selected at random, the recruitment sample was exclusively independent schools. Moreover, most schools who agreed to participate were from urban areas and were well-resourced and motivated to participate in a trial of a mental health program for their students. This may not accurately represent the population of Australian adolescents attending other types of schools and thus caution should be taken when generalising results to the broader population of Australian adolescents.

## Conclusion

In conclusion, sleep is an important risk factor to consider in the assessment and treatment of mental health in adolescents. For adolescents exposed to trauma, meeting nightly sleep duration guidelines may serve to protect against negative trauma-related symptoms such as anxiety and depression. Given the high prevalence of trauma exposure in adolescents, further research should prospectively collect data on representative samples to elucidate causal relationships between trauma, sleep and mental health outcomes. Understanding the role sleep plays in adolescents exposed to trauma may expose a critical pathway to mitigate the adverse effects associated with trauma exposure.

## Supplementary Information

Supplementary Material 1.

## Data Availability

The dataset generated and/or analysed during the current study are not publicly available due to conditions of ethical approval. Please contact the trial coordinator for more information (lucinda.grummitt@sydney.edu.au).
