# Supplementary material for: Sleep duration in adolescence buffers the impact of childhood trauma on anxiety and depressive symptoms
Source: BMC Public Health. 2025 Feb 4;25:437. doi: 10.1186/s12889-025-21621-x (PMC11792485; doi:10.1186/s12889-025-21621-x)
Supplement: Supplementary file 1 — Supplementary Material 1. [file 12889_2025_21621_MOESM1_ESM.docx]

**Supplement to Sleep duration in adolescence buffers the impact of childhood trauma on anxiety and depressive symptoms**

**Sleep Measures:**

**Sleep:**

**In general, over the last 3 months, approximately how many hours sleep per night did you get?**

- **Less than 5**
- **5**
- **6**
- **7**
- **8**
- **9**
- **10**
- **11**
- **More than 11**

**Do you have difficulty falling asleep?**

- - Yes
  - No

If yes, how severe is this difficulty?

| Mild | Moderate | Severe | Very Severe |
| --- | --- | --- | --- |

**Table 1. Trauma characteristics of participants in study, *n* = 752**

| Potentially traumatic events *^a^* | *Yes*  *n (%)* |
| --- | --- |
| Witnessing violence | 491 (67.3) |
| Victim of violence | 317 (43.5) |
| Sexual abuse | 98 (13.5) |
| Other forms of trauma exposure | 417 (57.4) |

*^a^* Missing data for 20 participants

**Table 2. Linear mixed effects models individually testing interaction effects between number of types of trauma exposures and sleep on mental health and well-being outcomes. All models adjusted for school clustering, gender and age.**

|  | Depressive symptoms | | Anxiety Symptoms | | Mental Wellbeing | |
| --- | --- | --- | --- | --- | --- | --- |
|  | B (SE) | *p* | B (SE) | *p* | B (SE) | *p* |
| Intercept | -3.37 (3.44) | 0.329 | 0.22 (3.38) | 0.949 | 31.86 (3.97) | **<0.001** |
| Number of trauma types | 1.40 (0.22) | **<0.001** | 1.50 (0.21) | **<0.001** | -0.97 (0.24) | **<0.001** |
| Does not meet sleep duration guidelines | 1.59 (0.65) | 0.015 | 1.29 (0.64) | 0.044 | -2.04 (0.73) | **0.005** |
| Number of trauma types*Does not meet sleep duration guidelines | 0.81 (0.30) | **0.007** | 0.49 (0.29) | 0.095 | -0.35 (0.33) | 0.295 |
| Intercept | 0.13 (3.26) | 0.967 | 2.87 (3.26) | 0.380 | 27.98 (3.91) | **<0.001** |
| Number of trauma types | 1.46 (0.19) | **<0.001** | 1.52 (0.19) | **<0.001** | -1.05 (0.22) | **<0.001** |
| Difficulties falling asleep | 3.30 (0.68) | **<0.001** | 2.40 (0.68) | **<0.001** | -2.10 (0.79) | **0.008** |
| Number of trauma types*Difficulties falling asleep | 0.43 (0.30) | 0.151 | 0.25 (0.29) | 0.403 | -0.13 (0.34) | 0.699 |

Note: Bold variables with p-value <0.05

**Table 3. Sensitivity analyses to test the influence of removing one item from the depression measure (PHQ-A) that measures sleep disturbances on associations between sleep measures and depressive symptoms.**

|  | Depressive symptoms | |
| --- | --- | --- |
|  | B (SE) | *p* |
| Does not meet sleep duration guidelines | 3.00 (0.37) | <0.001 |
| Difficulties falling asleep | 3.73 (0.37) | <0.001 |

**Table 4. Sensitivity analyses to test the influence of removing one item from the depression measure (PHQ-A) that measures sleep disturbances.**

|  | Depressive symptoms | |
| --- | --- | --- |
|  | B (SE) | *p* |
| Intercept | -4.26 (3.31) | 0.198 |
| Any trauma exposure | 1.94 (0.59) | **0.001** |
| Does not meet sleep duration guidelines | 0.42 (0.82) | 0.610 |
| Any trauma exposure*Does not meet sleep duration guidelines | 2.82 (0.90) | **0.002** |
| Intercept | -0.90 (3.21) | 0.779 |
| Any trauma exposure | 2.57 (0.53) | **<0.001** |
| Difficulties falling asleep | 2.91 (0.94) | **0.002** |
| Any trauma exposure*Difficulties falling asleep | 0.47 (1.01) | 0.643 |

Note: Bold variables with p-value <0.05

**Table 5. Sensitivity analyses to test the influence of removing one item from the trauma exposure measure (CTS) that asks about witnessing violence.**

|  | Depressive symptoms | | Anxiety Symptoms | | Mental Wellbeing | |
| --- | --- | --- | --- | --- | --- | --- |
|  | B (SE) | *p* | B (SE) | *p* | B (SE) | *p* |
| Intercept | -6.49 (3.60) | 0.072 | -2.19 (3.52) | 0.533 | 33.80 (4.02) | **<0.001** |
| Any trauma exposure | 2.49 (0.55) | **<0.001** | 2.71 (0.53) | **<0.001** | -2.01 (0.60) | **<0.001** |
| Does not meet sleep duration guidelines | 1.05 (0.69) | 0.126 | 0.51 (0.67) | 0.448 | -1.81 (0.75) | **0.016** |
| Any trauma exposure*Does not meet sleep duration guidelines | 3.22 (0.81) | **<0.001** | 2.72 (0.79) | **<0.001** | -1.43 (0.89) | 0.107 |
| Intercept | -2.30 (3.45) | 0.506 | 0.82 (3.43) | 0.811 | 29.48 (4.00) | **<0.001** |
| Any trauma exposure | 3.00 (0.49) | **<0.001** | 3.35 (0.48) | **<0.001** | -2.16 (0.55) | **<0.001** |
| Difficulties falling asleep | 3.59 (0.76) | **<0.001** | 2.75 (0.75) | **<0.001** | -1.93 (0.55) | **<0.001** |
| Any trauma exposure*Difficulties falling asleep | 0.95 (0.87) | 0.278 | 0.39 (0.86) | 0.650 | -0.68 (0.98) | 0.491 |

Note: Bold variables with p-value <0.05
